# Case of Abscess near the Axilla—Terminating with Profuse Hæmorrhage

**Published:** 1848-06

**Authors:** 


*Case of Abscess near the Axilla—Terminating with Profuse Hæmorrhage.*—I was requested to see a little girl four years old, with pain in her left arm. The upper third of the arm was very tender, upon pressure, but without any external evidence of inflammation. Upon taking hold of the limb and attempting to move it, the child would scream vehemently. The arm was held near to the side, the fore-arm thrown across the epigastrium, and the shoulder depressed, as in fracture of the collar bone. I could discover no evidence of fracture or dislocation. Rest and saturnine lotions were ordered. In three or four days a little redness appeared about an inch and a half below the axilla, under the arm. Poultices were directed—the child became more pale and wan—laid in its cradle, and took but little nourishment. The whole of the upper part of the limb became inflamed, and at the point where the first spot appeared, it began to soften; fluctuation was very apparent, and I was urged by the mother to open it. This I declined doing, without assigning any reason, and, indeed, without having any, except a disinclination to do so. Soon after leaving the house, I began to reflect whether I should not have done so, knowing that it would be followed by relief to my little patient. I hesitated, and was almost ready to turn back, to comply with the reasonable request of the mother. On my visit the next morning, the mother accosted me thus: "Doctor, you must lance this place, indeed you must." It was examined thoroughly, and was evidently fit to open. I had left my case at home and could not do it. The child suffered intensely, and the mother had been nearly worn out with twelve days of watching and anxiety. The poultices were continued, and anodynes were administered, and nourishing food directed. In the afternoon I was sent for in great haste. Not being at home at the time, I did not receive the message for an hour after it was left. On my return I went to the little sufferer; she lay pale cold, and nearly pulseless from excessive hæmorrhage. The abscess had broken, and when the discharge occurred, as the mother states, she attempted to raise the arm, and the blood jetted out in a stream, several feet across the room. Pressure was made above the orifice with the finger, the applications all removed, the arm made bare, and covered again with towels wet with cold water. Brandy and chicken broth were administered, but the stomach rejected them. Resort was then had to injections of beef tea, and sinapisms to the extremities. Carb. ammon. in emuls: amygdal: was administered, and retained. The pulse rose a little, and after tone was restored in some measure to the stomach, brandy and water were freely given. I deemed it useless to attempt to secure the artery, as the screams and struggles of the child might have increased the hæmorrhage, and as the orifice was too small to enter with the tenaculum or needle without a further division. Reliance was placed on the cold affusions, and perfect rest—they were sufficient. By pursuing this plan for several days, re-action was restored, and the patient is now recovering. Much thick and offensive matter was thrown of from the tumor, and continues, to some extent, at this time. The swelling has entirely subsided, and there remains an inability to extend the fore-arm from contraction of the biceps muscle, the radial tendon of which rises distinctly into the bend of the arm, when the extensor muscles attempt to act. The substance of the biceps muscle, and the coats of some one of the important branches of the brachial artery must have been embraced in the sphere of the suppurative action, and suffered a dissolution during its progress, in order to give rise to the results which followed. Had the abscess been penetrated by the lance, the hæmorrhage would, no doubt, have been attributed to its use; and had the patient died, the reflections of the operator must certainly have been very unsatisfactory.—*N. J. Med. and Surg. Rep.*

